# Supplementary material for: Documenting the implementation processes and effects of the data use initiatives in primary health care settings in Tanzania: A before-after mixed methods study protocol
Source: PLoS One. 2024 May 31;19(5):e0303552. doi: 10.1371/journal.pone.0303552 (PMC11142556; doi:10.1371/journal.pone.0303552)
Supplement: S1 File — (DOCX) [file pone.0303552.s001.docx]

**S1: Theory of change for deploying data use initiatives in the primary health care in Tanzania**

| **Determinants of data use** | **Intervention component** | \|  \| \| --- \| | **Outputs** | \|  \| \| --- \| | **Outcomes** | \|  \| \| --- \| | \|  \| \| --- \| |  |  |
| --- | --- | --- | --- | --- | --- | --- | --- | --- | --- | --- | --- | --- | --- |
|  |  |  |  |  | **short term outcome** |  | **intermediate outcomes** |  | **Long-term outcomes** |
| **Organizational determinants** |  |  |  |  |  |  |  |  |  |
| *Governance* | Recruit & equip data use coordinator |  | Resourceful and highly performing coordinator |  | Knowledge, skills and competences |  | Improved data use for decision making and planning |  |  |
|  | Develop and institutionalize guidelines to support data use |  | Availability of standardized data use guidelines |  | self-confidence and enhanced capabilities |  | Institutionalized data use culture at all levels |  | 1. Improved health system performance (patient centered care, service delivery and project management) |
|  | Facilitate use data in decision making and planning |  | Governance, management and frontline staff oriented on data use |  |  |  |  |  | 2. Improved health status (outcomes) |
|  |  |  | Governance and management structures use data in decision making and planning |  | consistency across sites and satisfaction |  | Improved data use for decision making at all levels |  |  |
| *Equipment and tools* | mapping of existing data use tools and identifying gaps |  | Availability of data use mapping reports |  | consistency across sites, simplicity, empowerment and satisfaction |  |  |  |  |
|  | Facilitate constant availability of data use equipment and tools |  | Availability of data use tools |  | Efficiency, accountability, capability |  | Improved data use for decision making at all levels |  |  |
|  |  |  | Existence of trained staff on the use of tools including PPM |  | Efficiency, accountability, capability |  |  |  |  |
| *Planning* | Facilitate inclusion of data use activities in comprehensive health plans |  | Existence of plans that have inclusion of data use issues |  | Capability, Accountability |  | Improved data use for decision making at all levels |  |  |
| **Technical determinants** |  |  |  |  |  |  |  |  |  |
| *Infrastructure(physical buildings, ICT systems)* | Construction/allocation of special room and installation of ICT systems for data use |  | Availability of equipped data use rooms |  | Motivation, opportunity possibility of discussing, analyzing and visualizing data, & satisfaction |  | Instituonalized data use culture |  |  |
| *HIS Design and operability* | Facilitate availability health information system (HIS) that have featured that support data use (interoperable, works both online and offline, has dashboard for visualization (e.g., score card), |  | Availability of HIS that support data use |  | Motivation, opportunity possibility of discussing, analyzing and visualizing data, & satisfaction |  | improved data quality |  |  |
|  | Develop simple self-learning HIS user’s manual |  | Resourceful and highly performing health care workers |  | self-confidence and enhanced capabilities |  |  |  |  |
|  | Facilitate Data quality check activities |  | \| Availability of quality data ready for use \| \| --- \| |  | self-confidence and enhanced capabilities |  | Improved data use for decision making and planning |  |  |
| **Behavioral determinants** |  |  |  |  |  |  |  |  |  |
| *Capability (knowledge, skills, competency inquiry, & problem solving in HIS tasks)* | Organize data use Forums at district level |  | Resourceful and highly performing health care workers |  | knowledge, skills, competency inquiry, & problem solving in HIS tasks |  | Improved data use for decision making and planning |  |  |
|  | Conduct trainings on data demand, data use and data manipulation |  | Governance, management and frontline staff knowledgeable, skilled and have positive attitude on data use |  | knowledge, skills, competency inquiry, & problem solving in HIS tasks |  | Improved data use for decision making and planning |  |  |
|  | Orientation on QI strategies on data use inquiry |  | number of trained personnel |  | knowledge, skills, competency inquiry, & problem solving in HIS tasks |  | ability to use of driver diagrams |  |  |
| *Opportunity* | orientation on organizational readiness for data availability and use |  | Availability of real time data |  | Knowledge, skills, Competences, self-confidence, self-esteem and capabilities |  | Data use in planning and in routine decision |  |  |
|  | Regular data review meeting (weekly to monthly) |  | \| Availability of quality data ready for use \| \| --- \| |  | Knowledge, skills, Competences, self-confidence, self-esteem and capabilities |  | Use of data in decision making and planning at all levels |  |  |
| *Motivation* |  |  |  |  |  |  |  |  |  |
|  | Promote accessibility of real time data at all levels (who actually accesses data and why?) |  | Availability of data in all sections in the organization/ facility |  | Efficiency, quality of delivered ,and competence |  | Institutionalized data use culture |  |  |
|  | Data use supportive supervision to promote accountability |  | Resourceful and highly performing health care workers |  | Efficiency, quality of delivered ,and competence |  | Improved data use for decision making and planning |  |  |
|  | Orientation to leaders at different levels on importance of data use in planning and decision making |  | Resourceful and highly performing leaders at all levels |  | Efficiency, quality of delivered ,and competence |  | Improved data use for decision making and planning |  |  |
|  | Facilitate accessibility of information related to health information system changes and troubleshooting |  | Availability of real time information and data |  | Efficiency, quality of delivered ,and competence |  | Data use for decision making and planning |  |  |
|  | Award best performers in data use at all levels |  | \| Motivated health workers / managers \| \| --- \| |  | \| Efficiency, quality of delivered ,and competence \| \| --- \| |  | Improved quality of data |  |  |
|  |  |  |  |  |  |  | \|  \| \| --- \| |  |  |
| **CONTEXTUAL FACTORS:** | | | | | | | | | |
| 1) intervention factors (comprehensiveness of intervention description, strategies to facilitate implementation, quality of delivery of the intervention), 2) Contextual factors (socio-cultural context, funding, partnerships, coordination and other interactions and 3) Stakeholder factors (responsiveness and personal context) 3)  Internet connectivity 4) Capacity of Central government and facilities to employ new staff 5) Consistency availability of electricity 6) Leadership dynamics | | | | | | | | | |
