# Supplementary material for: Documenting the implementation processes and effects of the data use initiatives in primary health care settings in Tanzania: A before-after mixed methods study protocol
Source: PLoS One. 2024 May 31;19(5):e0303552. doi: 10.1371/journal.pone.0303552 (PMC11142556; doi:10.1371/journal.pone.0303552)
Supplement: S2 File — (DOCX) [file pone.0303552.s002.docx]

S2: Data collection tools

**Tool # 1: STAR RATING TOOL FOR HEALTH FACILITIES**

**A: BACKGROUND INFORMATION**

- Region
- District
- Health facility

**B: HEALTH FACILITY INFORMATION**

- Facility Code (HFR)
- Facility Name (DHIS)
- Facility Location
- Facility Ownership Category
- Facility Owner (institution / authority)

**C: MANAGEMENT OF HEALTH FACILITY AND STAFF PERFORMANCE**

Health facility management

- Human resource availability at duty station
- Human resource management
- Functioning of the human resource management teams
- Functioning of the quality improvement teams

Facility Autonomy and Fiscal Decentralization

- Local level planning and budgeting
- Facility operating bank account
- Appropriate local expenditure on medicines and health products

Working conditions

- Housing or housing allowance for key staff
- On-call amenities
- Extra duty and on-call allowances budgeted

**D: USE OF FACILITY DATA FOR PLANNING AND SERVICE IMPROVEMENT**

Function of HMIS

- Staff trained on HMIS
- HMIS tools in use and filled correctly

Information use and dissemination

- Data interpreted and used at the facility
- Facility profile report shared with village and wards

Medical records

- Recording and retrieval of medical records
- Confidentiality assured for patient records

**E: ORGANISATION OF SERVICES**

**Service provider charter**

- Facility signage (name, working hours and on-call roster)
- Services, insurance benefits and charges are displayed
- Service charter on waiting times for core healthcare services
- Schedule for special clinics is displayed

Client Flow

- Optimal client flow
- Client waiting time is monitored
- Health promotion services
- Facility-based health education plan in place
- Outreach health promotion services are scheduled

**F: CLIENT FOCUS**

Client Service Charter

- Client service charter displayed
- Client service charter is monitored
- Client feedback mechanism and complaint handling

Client Satisfaction

Clients satisfied with services provided

**G: INFECTION PREVENTION AND CONTROL**

Infection Prevention Control (IPC)

- Health facility and surroundings are clean
- Antiseptic and disinfectants are available
- Accidental exposure to blood and body fluids handled
- Safe injection use
- Hand washing in all service areas
- Instrument sterilization or high-level disinfection
- Personal protective equipment used
- Laundry services
- Healthcare waste disposal

**Healthcare waste disposal facilities**

- Staff trained on healthcare waste management
- Waste segregation equipment and supplies

**H: QUALITY OF CARE**

**Reproductive, Maternal, Neonatal, Child and Adolescent Health (RMNCAH) Services**

- ANC services follow guidelines
- Family planning services follow guidelines
- Cervical Cancer screening is provided
- Immunization services follow guidelines
- The facility is able to perform CEmONC
- CEmONC training
- Checklists, charts, and protocols for management of pregnancy and childbirth available
- Partographs for mothers in labour are correctly filled and used
- Maternal death audits/reviews conducted as per MPDSR guidelines
- Availability of medicines and medical supplies for obstetric emergencies
- Post-natal and essential new born care.
- Child growth monitoring and HIV Exposed Infants (HEI) identification

**Nutrition**

- Personnel trained in nutrition service provision
- Guidelines, toolkits, job aids and IEC materials available for nutrition services
- Availability of anthropometric equipment and supplies for nutrition services
- Nutrition assessment, education and counselling follows practice guidelines
- Management of Severe Acute Malnutrition (SAM)

**Non-Communicable Diseases (NCD) and Mental Health Conditions**

- Facility provide services for non-communicable diseases (NCD) & Mental Health
- Staff trained and prepared for mental health and NCD service provision
- Facility readiness for NCD/mental health services
- Availability of medicines and medical supplies beyond the tracer items

**I: CLINICAL SUPPORT SERVICES**

**Pharmaceutical Service**

- Qualified pharmaceutical cadre
- Good dispensing practice
- Availability of essential health commodities
- Accountability for medicines issued to clients
- Inventory management per ILS guidelines
- Appropriate storage and handling of medication

**Laboratory services**

- Dedicated room for lab services
- Qualified laboratory cadre
- Essential laboratory tests provided as per minimum requirements of National Standards for Medical Laboratories
- Established turnaround time for results
- Quality assurance and control processes for tests
- Laboratory safety system in place
- Laboratory supplies management system in place
- Availability of required equipment to test or collect sample (for referral) for appropriate IDSR/Infectious diseases
- Availability of lab IDSR reporting system
- Availability of SOP for specimen collection, packaging and transportation

**Tool # 2: Questionnaire for health workers**

- Name of the region
- Name of the Council
- Name of Health facility

**SECTION I: BACKGROUND INFORMATION**

1. What is your current age..........................................( years)
2. What is your sex ( observe and record/) ; 1) Male 2) Female/
3. What is the highest level of education you have? /1) None 2) Some 3) completed primary 4) Some secondary 5) completed ordinary level 6) completed advanced level secondary 7)Post-secondary ( university, Diploma)
4. What is your cadre? 1) Clinician 2) Nurse 3) Laboratory 4) pharmacy / dispenser 7) others (specify).
5. What is your current marital status1) Single/Never married/No partner 2) Unmarried, With partner 3)Married (monogamous) 4) Married (polygamous) 5) Widowed 6) Separated (divorced )

SECTION II: GOVERNANCE FOR DATA USE

1. Does this facility has a data use coordinator/ focal person? 1. Yes 2. No
2. If yes, what are the responsibilities of the data use coordinator/ focal person
3. Are guidelines to support data use available at this facility?
4. If Yes, please name the guideline
5. If yes, are they are available in all sections of the facility?
6. Does your facility use evidence from data in decision making and facility planning?**?**
7. If yes, when does the facility (members of the facility) use data? SECTION III: EQUIPMENT AND TOOLS FOR DATA USE
8. Which tools for data use are available at the facility? ( mention)
9. Do you have a specific data reporting form?
10. Are data collection tools available all the time of the year?
11. Does your facility use GoTHOMIS?
12. If not, which electronic tool do you use for health information management?

SECTION IV: BEHAVIOURS FOR DATA USE

Please circle the word, which best describes what relates to you in relation to data use in this health facility for decision making and facility planning

|  |  | Strongly agree | Agree | Uncertain | Disagree | Strongly disagree |
| --- | --- | --- | --- | --- | --- | --- |
| 18 | I know that I have to use data while making decisions and planning at this health facility ] |  |  |  |  |  |
| 19 | I know the importance of using data in decision making and facility planning. |  |  |  |  |  |
| 20 | I have the required skills to use data generated at this health facility for decision making |  |  |  |  |  |
| 21 | I have the skills required for Data processing |  |  |  |  |  |
| 22 | I have skills required for data analysis |  |  |  |  |  |
| 23 | I have skills requited for data displaying |  |  |  |  |  |
| 24 | I know how to use the data collection tools available ( Ledgers, registers and tally sheets) at this facility / |  |  |  |  |  |
| 25 | I know how to use the electronic gadgets used to collect and process data ( digital devices, computers and data bases) |  |  |  |  |  |
| 27 | I am confident in using data in deciding for actions related to services I provide in this health facility |  |  |  |  |  |
| 28 | I have self-control to go on using data for decision making and planning at this health facility |  |  |  |  |  |
| 29 | I have access to all data required for decision making and planning |  |  |  |  |  |
| 30 | I can access to all the data required for decision making for services I provide and planning at any time I need? |  |  |  |  |  |
| 33 | In this facility the management team supports the use of data in decision making and facility planning |  |  |  |  |  |
| 34 | In this facility the health facility governing committee supports the use of data in decision making and facility planning |  |  |  |  |  |
| 35 | The CHMTs takes data use as an important component during supportive supervision in this health facility |  |  |  |  |  |
| 36 | Using data in clinical/ administrative decision making is part of the routine activities in this health facility |  |  |  |  |  |
| 37 | Using data in facility planning is part of the routine activities in this health facility |  |  |  |  |  |

SECTION V: CONTEXTUAL FACTORS

Indicate your agreement with the following statements

|  |  | Strongly agree | Agree | Uncertain | Disagree | Strongly disagree |
| --- | --- | --- | --- | --- | --- | --- |
| 38 | This place has internet connectivity problem |  |  |  |  |  |
| 39 | This health facility has adequate staff |  |  |  |  |  |
| 40 | The health facility leadership supports data use initiatives |  |  |  |  |  |
| 41 | Organization culture does not support data use initiatives |  |  |  |  |  |

**Tool #3: Exit interview questionnaire**

1. Name of the Region: 1) Dodoma 2) Tanga 3)Dar es salaam 4) Mwanza
2. Name of the District Council 1) Dodoma MC 2) Chamwino 3)Tanga CC 4)Korogwe DC 5)Temeke 6)Kinondoni 7)Mwanza MC 8) Misungwi
3. Ward……………
4. Village/Street …………
5. Name of the Health Facility
6. Type of Health Facility 1) Dispensary 2) Health center

SECTION A: DEMOGRAPHIC INFORMATION

1. Sex 1) Female 2) Male
2. How old are you?............... (years)
3. Marital status 1) Married 2) single
4. Highest level of education 1) Primary 2) Secondary 3) Post secondary 4) Never went to school
5. What is your source of income? ……….( Mention )
6. What is the size of your household?.....................( number of people)
7. How many times have you received services at this Health facility?.......................
8. How much distance do you have to take in order to reach health care? …………………(km)
9. What department did you receive services today? 1) OPD 2) IPD 3) RMNCAH 4) CTC
10. How did you cover the cost of your health services today? 1) Out of pocket 2) Insurance 3) waiver and Exemption

**Other information from client**

1. Was the waiting time acceptable to you? 1) Yes 0) No
2. Did the health worker examine you? 1) Yes 0) No
3. Did the health worker explain about your care, or illness, and about any tests or treatment? 1) Yes 0) No
4. Did you receive all the prescribed medicines? 1) Yes 0) No
5. Did you understand how to take the medicines? [probe] 1) Yes 0) No
6. Were the health workers polite and respectful? 1) Yes 0) No
7. Did you have enough privacy during your visit? 1) Yes 0) No
8. Did you find the facilities clean and in order? 1) Yes 0) No
9. Are the fees and charges fair and affordable to you? [Q also applies to CHF/ NHIF members] 1) Yes 0) No
10. Was your visit satisfactory overall? 1) Yes 0) No
11. Please explain why not satisfactory
12. What would you like to see improved?

**Prompt to Attention**

1. How often did the health service providers listen to what you said with full attention during provision? 0)Never happens 1)Slightly Often 2) Often 3)Very often
2. How often your statements were deeply understood by the health service providers? 0)Never happens 1)Slightly Often 2) Often 3)Very often
3. How often did health service providers spend enough time in asking you questions? 0)Never happens 1)Slightly Often 2) Often 3)Very often
4. How often the health service providers were accurately and actively in following up your treatment process? 0)Never happens 1)Slightly Often 2) Often 3)Very often
5. Are the clients with similar needs treated equally in this health facility? 0)Never happens 1)Slightly Often 2) Often 3)Very often
6. Are Clients with un equal needs treated equally in the health units? 0)Never happens 1)Slightly Often 2) Often 3)Very often
7. Has the health facility always met your expectations? 0)Never happens 1)Slightly Often 2) Often 3)Very often

**Respect to Dignity**

1. How often did the health service providers show courtesy and affection towards you during service provision? 0)Never happens 1)Slightly Often 2) Often 3)Very often
2. How often did the health care workers paid attention specifically into your needs and characteristics? 0)Never happens 1)Slightly Often 2) Often 3)Very often
3. How often is respect shown for the client’s desire for privacy during treatment and examination? 0)Never happens 1)Slightly Often 2) Often 3)Very often

**Clear Communication**

1. How often did health care workers explain things in a way you could understand? 0)Never happens 1)Slightly Often 2) Often 3)Very often
2. How often health care workers explain things and issues related to your health in detail for you? 0) Not satisfied 1) Satisfied 2) Very satisfied 3) Highly satisfied
3. How would you rate your experience about how well you were treated as human during interaction with nurses? 0) Not satisfied 1) Satisfied 2) Very satisfied 3) Highly satisfied
4. How would you rate your experience about how well you were treated as human during interaction with laboratory staff? 0) Not satisfied 1) Satisfied 2) Very satisfied 3) Highly satisfied
5. How would you rate your experience about how well you were treated as human during interaction with medical doctors/clinicians? 0) Not satisfied 1) Satisfied 2) Very satisfied 3) Highly satisfied
6. How would you rate your experience about how well you were treated as human during interaction with security staff? 0) Not satisfied 1) Satisfied 2) Very satisfied 3) Highly satisfied

1. How would rate overall quality of interaction at this health facility? 0) Not satisfied 1) Satisfied 2) Very satisfied 3) Highly satisfied

Autonomy

1. How big a problem if any was it to get an appointment with the health care worker of your choice? 0) Very big 1) Big 2) Average 3) No problem
2. How big a problem (if any) was it to use other health facility other than the one you usually go to? 0) Very big 1) Big 2) Average 3) No problem
3. Do you feel that physicians provide you with choices and options on the services offered? 0) Very big 1) Big 2) Average 3) No problem

**Access to Care**

1. How long did you have to wait to get medical consultation from service provider? ……minutes
2. How long did you have to stay in the waiting room..............minutes
3. How long did you have to stay at the pharmacy or dispensing area?……(minutes)
4. How long did you have to stay waiting for laboratory services and results?.................( minutes)

**Confidentiality**

1. How often your interviews remain confidential? 1) Never happens 2) Slightly Often 3) Often 4) Very often
2. Do Health care workers keep your personal information and records confidential? 1) Never happens 2) Slightly Often 3) Often 4) Very often
3. Is the confidentiality maintained in this health facility? 1) Never happens 2) Slightly Often 3) Often 4) Very often

**Basic Amenities**

1. I agree that this health facility has enough buildings for service delivery 1) Strongly agree 2) disagree 3) Agree 4) strongly agree
2. I agree that this facility has enough staff to service clients 1) Strongly agree 2) disagree 3) Agree 4) strongly agree
3. I agree with the quality of direction aids of this facility 1) Strongly agree 2) disagree 3) Agree 4) strongly agree
4. I agree with the cleanliness of this surroundings 1) Strongly agree 2) disagree 3) Agree 4) strongly agree
5. I agree with the waiting environment of this facility (waiting seats) 1) Strongly agree 2) disagree 3) Agree 4) strongly agree
6. Are you convenient with the safety of service delivery environment in this facility? 1) Strongly agree 2) disagree 3) Agree 4) strongly agree
7. Is there access to clean water in this health care facility? 1) Strongly agree 2) disagree 3) Agree 4) strongly agree
8. Do the cleanliness of the toilets in the health facility maintained? 1) Strongly agree 2) disagree 3) Agree 4) strongly agree
9. Are there facilities for people with disabilities in the health care? 1) Strongly agree 2) disagree 3) Agree 4) strongly agree
10. There is adequate space in the rooms of this facility 1) Strongly agree 2) disagree 3) Agree 4) strongly agree
11. The bed sheets of this facility are usually clean? 1) Strongly agree 2) disagree 3) Agree 4) strongly agree
12. Is the smell in this health care facility pleasant? 1) Strongly agree 2) disagree 3) Agree 4) strongly agree

Tool # 4: **Observation checklist**

**A: Background information**

Checklist #……**01**……

1. District
2. Name of the health Facility
3. Level of Health Facility
4. Catchment population
5. Women of reproductive age population
6. Adolescents (12-24years) population
7. Children under five years population
8. Number of health care workers
9. Number of Community Health care workers
10. Staff training schedule
11. Staff annual leave roster
12. Sources of funds allocated
13. Trend of fund disbursement
14. Number of buidings
15. Number of vehicles
16. Internet service provider
17. Number of annual OPD attendance
18. 18.Number of ANC, attendance

19. Number of people accessing family planning

20. Immunization coverage

**B: Data use information**

| No | Observed Item | Present | |
| --- | --- | --- | --- |
|  |  | Yes | No |
| 1 | Presence of data coordinator |  |  |
| 2 | Formal letter articulating duties and responsibilities of data coordinators |  |  |
| 3 |  |  |  |
|  | Training report on data use seminar provided to health workers |  |  |
|  | Availability of summarized data use forms for use in governance and management meetings |  |  |
|  | Availability of summary of data form used by the facility during the comprehensive planning process |  |  |
|  | Availability of equipment facilitating data use in facilitating  Computer Internet infrastructure Special room for data use Budget for internet |  |  |
|  | Availability of summary of weekly/monthly/quarterly data used by the facility |  |  |
|  | QI reports that have data use part |  |  |

C: Data use tools

| No | Observed Item | Present | |
| --- | --- | --- | --- |
|  |  | Yes | No |
| 1 | Availability of data use SOPs/ guidelines |  |  |
| 2 | Presence of ICT tools to support data use |  |  |
| 3 | Presence of data use equipment ( name them) |  |  |
| 4 | Presence of data sources  Electronic? FFARS   - eLMIS - Planrep - GoTHOMIS - Others (Specify) - GePG - MUSE - HRHIS - WISN/POA - eIDSR   Manual HIMS books Registers   - Tally Sheets - Reports |  |  |
|  | Data use meeting minutes ( DQA) |  |  |

Tool #5: **In-depth interview guide**

**Section A: Governance guide**

1. What responsibilities does the data use coordinator play in this facility?
2. In what specific areas do service providers use data to decide for your facility?
3. What are the factors which influence service providers to use data in your facility?
4. What are the necessary infrastructures required by the facility to facilitate data use?
5. How do you recognize a facility plan which was informed by data?
6. How your facility has institutionalized data use in management and governance? /
7. In what aspects of the planning process do health workers use data?

**Section B: Technical guide**

1. What should the equipped data use facility look like?
2. How do you recognize a HIS that facilitate data use in health facility?
3. What are the key elements would you like a friendly use of HIS to have?
4. What are the key issues necessary for health service providers to easily use and own HIS in their facility?

**Section C: Behavior guide**

1. What are the key orientations/behavioral aspects necessary for stimulating data use culture?
2. How do you identify data use culture in the health facility?
3. How do QI Teams influence data use culture in health facilities?
4. What are the strategies employed by the health facilities to facilitate data use culture?
5. How do you or your health facility identify and ensure the facility has real-time data?

**Section D: Contextual Factors**

1. What should the government put in place to ensure health service providers use data willingly?
2. What should the stakeholders do to stimulate data use in the facility decision-making process?
3. What are the contextual factors limiting or facilitating data use in decision-making at your facility?

**Tool #6: DHS2 data extraction checklist.**

1. District
2. Name of the health Facility
3. Level of Health Facility
4. Number of health facility deliveries
5. Number of pregnant mothers completed 4th ANC visit,
6. ANC Total Pregnant Women (PW) tested,
7. Number of pregnant women who tested positive for HIV (First Test),
8. Proportion of pregnant women on ARV to prevent HIV transmission
9. Number of Maternal Deaths
