# Supplementary material for: Documenting the implementation processes and effects of the data use initiatives in primary health care settings in Tanzania: A before-after mixed methods study protocol
Source: PLoS One. 2024 May 31;19(5):e0303552. doi: 10.1371/journal.pone.0303552 (PMC11142556; doi:10.1371/journal.pone.0303552)
Supplement: Attachment — Submitted filename: Response to Reviewers.docx [file pone.0303552.s003.docx]

Department of Public Health, St. Francis University College of Health and Allied Sciences, P.O. Box 175, Ifakara, Tanzania

Email: kaloloa@gmail.com

22/09/2023

**The editor, PLOS ONE journal ,**

Please find enclosed revised manuscript : Documenting the implementation processes and effects of the data use initiatives in primary health care settings in Tanzania: A before-after mixed methods study protocol; which we would like to be considered for publication in your journal. In this letter we express our gratitude to the editorial team and reviewers for commenting on our paper. We also provide responses to the minor comment raised by reviewers as detailed below ( Table 1). We indicate the changes in the manuscript by highlighting the changes in track changes . The competing interest statement is updated to : “Jennie Jaribu works with UNICEF, Tanzania. Other authors declare no competing interests” .This does not alter our adherence to  PLOS ONE policies on sharing data and materials. The amended funding statements is updated to “ “The authors wish to thank the teams from Data.Fi, UNICEF, CERHI, PORALG and the councils participating in the data use intervention for their inputs during the preparation of the theory of change”. The supplementary files have been removed from the main manuscript . Reference list has been reviewed to ensure completeness and correctness

I as the first author will act as a corresponding author and deal with all pre-publication commitments. I confirm that the paper has not been nor will not be submitted elsewhere.

I look forward to your feedback

Dr Albino Kalolo

Table 1: Response to reviewer comments

| S/N | Comment | Response |
| --- | --- | --- |
|  | Reviewer #1 |  |
| 1 | How was the sample size calculated in the component aiming at establishing the effects of data use initiatives? | Thank you for your comment. The description of sample size for each data collection tool ( method) has been described in the manuscript ( Lines 295-326, taking into account the mixed methods nature of the study) |
| 2 | Provide a brief description of the data use initiatives so as to allow for understanding what the standard you are measuring the process of implementation against is and provide a reference | The description of the components of the data use initiative are described in line also with the theory of change |
|  | Reviewer #2 |  |
| 1 | For research protocols always use futuristic tense "will be", lines 144 and 145 | Thank you for the observation , this has been rectified accordingly |
| 2 | Line 122: "fastness" instead of "fidelity" | Thank you for your comment, our aim is to check whether the initiative will be implemented as intended that is ” Fidelity” |
| 3 | Line 125: "unravel" instead of "disentangle" | Thank you , this has been changed |
| 4 | Line 212: Mention the contextual influences | Thank you, we have included examples of contextual factors |
| 5 | Line 213: "The study will adopt the following steps" instead of "we will follow the following steps" | Thank you , this has been changed |
